# Fabrication of Hybrid Coated Microneedles with Donepezil Utilizing Digital Light Processing and Semisolid Extrusion Printing for the Management of Alzheimer’s Disease

**DOI:** 10.1021/acs.molpharmaceut.4c00377

**Published:** 2024-08-20

**Authors:** Paraskevi-Kyriaki Monou, Eleftherios G. Andriotis, Eirini Saropoulou, Emmanouil Tzimtzimis, Dimitrios Tzetzis, Georgios Komis, Chrysanthi Bekiari, Nikolaos Bouropoulos, Efterpi Demiri, Ioannis S. Vizirianakis, Dimitrios G. Fatouros

**Affiliations:** † Department of Pharmacy, Division of Pharmaceutical Technology, Aristotle University of Thessaloniki, Thessaloniki 54124, Greece; ‡ Center for Interdisciplinary Research and Innovation (CIRI-AUTH), Thessaloniki 57001, Greece; § Digital Manufacturing and Materials Characterization Laboratory, School of Science and Technology, International Hellenic University, 14km Thessaloniki - N. Moudania, Thermi GR, Thessaloniki 57001, Greece; ∥ Department of Botany, School of Biology, Aristotle University of Thessaloniki, Thessaloniki 54124, Greece; ⊥ Faculty of Veterinary Medicine, School of Health Sciences, Aristotle University of Thessaloniki, Thessaloniki 54124, Greece; # Department of Materials Science, University of Patras, Rio, Patras 26504, Greece; ∇ Department of Plastic Surgery, Medical School, Papageorgiou Hospital, Aristotle University of Thessaloniki, Ag. Pavlos, Thessaloniki 56429, Greece; ○ Department of Pharmacy, Laboratory of Pharmacology, Aristotle University of Thessaloniki, Thessaloniki 54124, Greece; ◆ Department of Life and Health Sciences, University of Nicosia, Nicosia CY-1700, Cyprus

**Keywords:** 3D printing, microneedles, skin delivery, Alzheimer’s disease, transdermal delivery

## Abstract

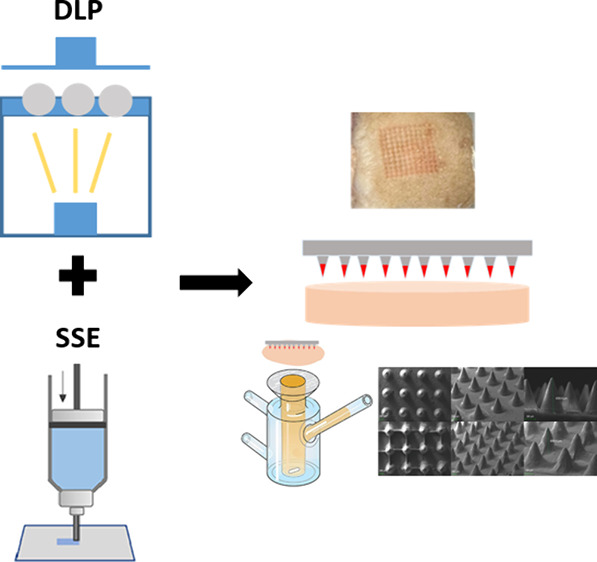

Microneedle (MN) patches are gaining increasing attention as a cost-effective technology for delivering drugs directly into the skin. In the present study, two different 3D printing processes were utilized to produce coated MNs, namely, digital light processing (DLP) and semisolid extrusion (SSE). Donepezil (DN), a cholinesterase inhibitor administered for the treatment of Alzheimer’s disease, was incorporated into the coating material. Physiochemical characterization of the coated MNs confirmed the successful incorporation of donepezil as well as the stability and suitability of the materials for transdermal delivery. Optical microscopy and SEM studies validated the uniform weight distribution and precise dimensions of the MN arrays, while mechanical testing ensured the MNs’ robustness, ensuring efficient skin penetration. In vitro studies were conducted to evaluate the produced transdermal patches, indicating their potential use in clinical treatment. Permeation studies revealed a significant increase in DN permeation compared to plain coating material, affirming the effectiveness of the MNs in enhancing transdermal drug delivery. Confocal laser scanning microscopy (CLSM) elucidated the distribution of the API, within skin layers, demonstrating sustained drug release and transcellular transport pathways. Finally, cell studies were also conducted on NIH3T3 fibroblasts to evaluate the biocompatibility and safety of the printed objects for transdermal applications.

## Introduction

Additive manufacturing, particularly 3D printing, plays a crucial part in creating personalized MN devices carefully constructed according to a 3D digital design. These designs incorporate specific attributes with accuracy, including needle length, shape, device thickness, density, and size, carefully tailored to meet the unique requirements of patients and the specific demands of the application site.^1^ This manufacturing technique, in contrast to traditional production methods, is both cost-effective and swift, allowing personalized combination treatments involving multiple APIs.^2^ MNs can be fabricated using various 3D printing methods, including extrusion-based techniques,^3^ as well as photopolymerization-based methods such as stereolithography (SLA) and digital light processing (DLP).^4,5^ Semisolid extrusion (SSE) 3D printing involves deposition of a gel or paste in sequential layers to create a 3D object. Upon extrusion, the material solidifies, providing support for the layers above.^6^ Additionally, the solvent casting procedure can be combined with semisolid extrusion 3D printing. This well-established technique includes dissolving a polymer in an organic solvent, resulting in the formation of films after drying.^7^ In the present study, two 3D printing processes, DLP and SSE printing, were combined to develop coated MN arrays, for the transdermal delivery of donepezil (DN) a cholinesterase inhibitor for the treatment of Alzheimer’s disease.

The World Alzheimer Report 2018 reveals that 70–80% of the 50 million individuals globally affected by general dementia are diagnosed with Alzheimer’s disease. Projections for 2050 are disturbing, indicating a significant increase to 152 million cases, signifying a substantial and pressing concern for societies worldwide.^8^ The rising number of Alzheimer’s disease patients has become an enormous burden for national healthcare systems. Therefore, improvement of current medication treatments is considered a necessity. The causes and pathogenic mechanisms of Alzheimer’s disease have not yet been fully discovered and the existing treatments offer relief from the symptoms of the disease.^9^ The patients require constant care due to clinical symptoms, including dementia and a decline in cognitive and verbal abilities. This places a substantial financial burden on both their families and society.^10^ Hence, the optimal approach for patients to attain a high-quality life entails the utilization of approved treatments. DN reversibly inhibits acetylcholinesterase, thereby it increases the levels of the neurotransmitter acetylcholine, with the aim to enhance mental and functional abilities and alleviate psychological symptoms.^11,12^

Donepezil (DN) is administered orally, causing many side effects, namely, nausea, diarrhea, vomiting, headaches, fatigue, and musculoskeletal problems that are mainly due to the unconditional attachment of the drug to peripheral receptors.^13,14^ These adverse effects have led to further research into alternative administration routes. The transdermal route has been investigated for DN delivery using nanofiber patches^15^ and lipid gels.^16^ Dissolving and hydrogel MNs have also been developed to enhance the penetration of DN through the skin.^17,18^ The aim of this study is to propose a novel transdermal drug delivery system using two different 3D printing processes and allowing the rapid manufacture of customized MN devices in a controlled and fully automated way. The developed MNs can easily adapt to the special needs of each patient regarding their shape, length, and drug dosage. In this way, they enhance the overall experience of patients with their treatment and facilitate the personalization of each device in a fast and easy way.

Transdermal drug delivery has gained significant attention as a viable, noninvasive route of administration.^19^ This is attributed to the substantial benefits it provides in comparison to oral delivery and hypodermic injections. Oral administration presents challenges, including drug metabolism in the liver, known as the first-pass effect, leading to potential side effects and degradation of the actives in the gastrointestinal tract.^20^ Moreover, elderly individuals, particularly those dealing with chronic neurological disorders, often encounter difficulties in swallowing, presenting a significant hurdle in their ability to take medication.^21^ Transdermal drug delivery may overcome the challenges presented by insoluble compounds, which typically result in poor absorption and reduced bioavailability during oral administration.

Hypodermic injections allow direct drug delivery into the systemic circulation, but they can be painful and triggering for certain patients with needle phobias, resulting in poor patient compliance.^22^ However, transdermal drug delivery systems are remarkably user-friendly, even for pediatric and geriatric populations, in contrast to injections, which necessitate administration by medical professionals and are invasive.^23^ The skin offers a large surface (20000 cm^2^), making it the biggest entrance to the human body.^24^ It consists of three main layers, the epidermis, the dermis, and hypodermis. The outermost layer, the epidermis, encompasses the stratum corneum (SC), with a thickness ranging from 10 to 20 μm, which poses the main barrier for drug absorption.^25,26^ Consequently, only a limited number of drugs with moderate lipophilicity, low molecular weight, and high potency can breach this barrier and reach the systemic circulation.^27^

To enhance skin permeability, diverse chemical, biochemical, and physical studies have been conducted.^28^ These methods include the creation of supersaturated drug solutions,^29^ eutectic mixtures,^30^ microdermabrasion,^31^ and the application of permeation enhancers.^32^ Microneedles (MNs) are an exceptionally efficient method for enhancing transdermal drug delivery, as they are able to bypass the SC barrier in a painless way.^33−36^ Generally, MNs can be divided into four distinct classifications: solid, drug-coated, dissolving, and hollow ones.^37^ In the present study, coated MNs were fabricated by combining two different 3D printing processes, DLP printing and semisolid extrusion. The produced MNs were evaluated with regard to their physiochemical and mechanical behavior. Imaging techniques were employed to better visualize the arrays and also to assess the permeation profile of the API. The toxicity was also examined by conducting cell studies and histological and immunohistochemistry tests in human skin samples before and after MN application. All the results indicated that these two 3D printing processes are able to produce coated MN arrays for skin delivery of actives in a safe and reliable way.

## Materials and Methods

### Materials

Biocompatible Class I resin (Dental SG) was purchased from Formlabs (Somerville, Massachusetts, United States). Isopropyl alcohol (IPA, 99.9%) and donepezil (DN) were purchased from Sigma-Aldrich (Darmstadt, Germany). Hydroxypropyl methylcellulose (Affinisol HPMC HME 15LV) was kindly donated by Dow Chemical Company (Midland, USA). All other reagents were of analytical grade.

### Methods

#### Production and Coating Method for the Produced MN Patches Using Two 3D Printing Processes

MN arrays were designed using AutoCAD 2019 (Autodesk Inc., CA, USA), exported as stl. files and loaded into the printer software for slicing. The needles were designed conical with a 0.5 mm base diameter and 1 mm length. The arrays consisted of 11 × 11 needles on a 20 × 20 × 1 mm circular substrate. Their dimensions were selected according to the literature. Generally, the MN’s dimension varies between the studies published.^38−41^ Typically, their sizes are varying between 25 and 2500 μm in length, 20 to 250 μm in width, and 1 to 25 μm in tip diameter.^42^ Using CAD software, the design was adapted to the needs of the present study. The MNs were printed using a DLP printer (PartPro x100, XYZ, Taiwan) loaded with the Dental SG resin to ensure the biocompatibility of the printed arrays for drug delivery. After printing, the MN patches were washed with IPA and cured for 60 min to ensure complete polymerization of the resin. An extrusion-based 3D Bioprinter (CELLINK Inkredible, Gothenburg, Sweden) was used for the coating process. A coating solution was prepared using 7% w/v HPMC in ethanol. After complete dissolution of the HPMC, DN was added at a final concentration of 8.5 mg/mL. After DN was dissolved, the ink solution was loaded into a syringe and then placed into the printer. Square films were designed by using AutoCAD and loaded into the printer software for slicing. The arrays were placed on the building platform and calibrated to ensure that the nozzle deposits the drug-loaded ink onto the needles as depicted in Figure 2. Upon coating, the MNs were left at ambient temperature overnight for solvent evaporation, and a thin film of HPMC containing the API was formed onto the needles.

### Physiochemical Characterization of the Coating Film

The coating material was evaluated using Fourier-transform infrared (FTIR) analysis, differential scanning calorimetry (DSC), thermogravimetric analysis (TGA), and X-ray diffraction (XRD) to further characterize the film and the interactions between the API and the polymers. FTIR analysis was conducted using the IR Prestige-21 instrument (Shimadzu, Kyoto, Japan), in the range of 650–4000 cm^–1^ with a resolution of 2 cm^–1^. A DSC 204 F1 Phoenix equipment (Netzsch, Selb, Germany) was employed to study the thermal behavior of all of the materials. The samples (5 mg) were placed in aluminum pans and heated to 200 °C, with a heating rate of 10 °C/min. TGA analysis (Shimadzu TGA-50, Tokyo, Japan) was also performed to fully characterize the thermal properties of the produced formulations. The samples were heated to 300 °C, under a nitrogen atmosphere with a heating rate of 10 °C/min. The XRD patterns were obtained using a D8-Advance instrument (Bruker, Germany) to evaluate the crystallinity of the tested materials. The diffractograms (Cu–Kα1; 40 kV, 40 mA) were recorded over the 2θ range of 5°–50° (step size, 0.02°; scanning speed, 0.35 s/step).

An *in vitro* release study was also conducted to examine the release of DN from the formulation coated on the needles. This study was carried out in PBS (pH 7.4) at 37 °C, and the release was monitored for 6 h. The coated MNs were immersed in glass vessels containing 50 mL of PBS, and aliquots were extracted at predetermined time points, centrifuged (4,000 rpm, 10 min), and analyzed by high-performance liquid chromatography (HPLC). The experiment was conducted in triplicate.

### Quantification of DN

DN was quantified using an HPLC system composed of a pump (LC-10 AD VP), an autosampler (SIL-20A HT), and an ultraviolet–visible detector (SPD-10A VP, Shimadzu, Kyoto, Japan). The mobile phase consisted of potassium dihydrogen phosphate buffer, 0.05 M, pH 2.3: acetonitrile (60:40% v/v), pumped at a flow rate of 1 mL/min. A Discovery HS C18 column (150 mm, 4.6 mm, 5 μm) was used as a stationary phase at ambient temperature and the analysis of DN was performed at 230 nm, while the injection volume was set at 30 μL. Standard samples of DN were analyzed in the concentration range 0.1–50 μg/mL (*R*^2^ = 0.999).

### Morphological Characterization of the Printed MNs

After printing, the MNs were characterized regarding their surface morphology and dimensions, using an optical microscope (Celestron MicroDirect 1080p HD Hand-held Digital Microscope, Celestron, Torrance, California, USA) and SEM (Zeiss SUPRA 35VP, Zeiss, Oberkochen, Germany). Their dimensions were measured using ImageJ (NIH, Bethesda, MD, USA) to ensure good printability of the MNs. Moreover, all of the arrays were weighed before and after coating to verify that the two processes can produce MN arrays with no variability in their dimensions or in their dosage accuracy.

### Mechanical Properties and Insertion Test

The printed MNs were subjected to compression and insertion tests, using a testometric machine (M500–50AT Testometric Company, Rochdale, UK), to evaluate their ability to penetrate the human skin without breaking or creating fragments that could infect the patient’s skin. For the compression test, the arrays were attached to a metal rod with double adhesive tape that was programmed to descend at a rate of 0.5 min/mm onto a metal plate until the MNs fail due to compression. The forces applied were significantly higher (300 N) than the forces applied during skin insertion (10–40 N). For the insertion test, the MNs were attached to a movable cylindrical probe and inserted into porcine skin at a speed of 0.5 mm/min with a force of 40 N applied for 30 s. After the test, the porcine skin was examined regarding the presence of perforation sites owned by the needles. All of the experiments were conducted in triplicate.

### Delivery Efficiency of DN

The ability of the needles to deliver effectively the API across the skin was assessed by using artificial skin. MN arrays (*n* = 3) were manually inserted into the artificial skin, which was prepared in the lab using agarose, according to the literature.^43^ After 5 min, the MNs were removed and the artificial skin was immersed in 20 mL of PBS and vortexed for 10 min. The amount of DN inside the samples was quantified using HPLC and the delivery efficiency was calculated according to the following equation:
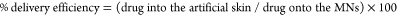


### Permeation Studies and Tape Stripping

Permeation studies were conducted to evaluate the ability of the printed MNs to deliver DN across the skin. For the in vitro permeation studies, human skin was obtained from cosmetic surgery. The skin was then mounted between the donor and the acceptor of Franz cells to evaluate the permeability of the active site with and without MNs. The acceptor was filled with PBS, pH 7.4, and the system was kept at 32 ± 1 °C throughout the experiment. The permeability of DN with and without piercing was monitored for 24 h. The coated MNs were manually inserted into the skin (*n* = 3) and they were removed after 5 min, while the coating film was applied onto it for the whole experiment (*n* = 3). At predetermined time points, aliquots of 0.5 mL were removed from the acceptor, and the amount of DN was quantified using HPLC.

At the end of the experiment, the Franz cells were disassembled, and the stratum corneum (SC) was removed from the skin samples using tape stripping to further quantify the amount of the API deposited onto it. To remove the SC, 20 adhesive tapes were cut in the same dimensions as the skin samples (2 cm in length), and they were pressed onto them using a roller to have a constant pressure at approximately 15 kp/cm^2^ (the same individual performed this procedure consistently throughout the study). Every strip had different directions and was rapidly removed from the skin for quantification. The strips were placed in 2 mL of methanol according to the following sequence: vial 1 (strip 1), vial 2 (strips 2–3), vial 3 (strips 4–5), vial 4 (strips 6–8), vial 5 (strips 9–12), vial 6 (strips 13–16), and vial 7 (strips 17–20). Subsequently, the vials were sonicated for 1 h to extract the API and centrifuged (10 min, 10000 rpm) prior to HPLC analysis (*n* = 3). To ensure that the tapes do not retain any drug content, the tapes were spiked with known concentrations of DN and subjected to the same sonication and centrifugation protocols. After HPLC analysis, the extraction efficiency was 98.7 ± 1.1%.

### Confocal Laser Scanning Microscopy (CLSM) Studies

To investigate the distribution of the API after MN penetration, we conducted confocal laser scanning microscopy (CLSM) studies were conducted. The produced MNs were coated with HPMC solution containing Nile Red (0.05 mg/mL) in ethanol. Nile Red (log *P* of 3.8) was selected as a substitute for DN (log *P* of 4.3). After solvent evaporation, the needles were inserted into human skin samples, mounted onto the Franz cells, for 5 min. At predetermined time points, the skin specimens were removed from the cells and immersed in liquid nitrogen. To investigate the distribution of the dye in the different skin layers, the specimens were cut from the dermis to SC using a cryo-microtome (Reichert-Jung Cryo microtome 1206, with cooling aggregat Frigomobil, Labexchange, Burladingen, Germany), with the thickness of 40 μm. 10 slices and 3 areas from each specimen were investigated using Zeiss LSM780 CLSM (Zeiss, Oberkochen, Germany) equipped with a 40 × /1.3 NA oil immersion lens, to determine the distribution of Nile red into the skin. The specimens were mounted onto positively charged glass slides prior to analysis.

### Histological Evaluation and Immunohistochemistry

To assess the morphological characteristics of the skin and its integrity after piercing with the MNs, histological and immunohistochemical evaluations were conducted. At the end of the permeation experiment, the skin samples were treated with 10% formaldehyde solution before embedding them in paraffin. Sections of 10 μm thickness were cut and stained with hematoxylin eosin dyes (H&E) or against antigens of interest.

To evaluate specific histological traits of MN-treated and untreated skin samples, one out of every 10 serial sections was stained with H&E. On average, 10 sections per skin sample were stained and subsequently analyzed under an optical microscope (Nikon Eclipse 80i microscope, Nikon Europe B.V., Amsterdam, Netherlands). Double immunofluorescence staining for pancytokeratin (pCK), expressed by skin epithelial cells, and fractin (FRA), an accurate apoptotic marker, was conducted to assess the integrity of the skin after MN application and to ensure the safety of the produced MN arrays. Sections of the above-mentioned paraffin-embedded skin samples were further processed for immunohistochemical (IHC) analysis to detect the expression of the epithelial marker pancytokeratin (pCK) and the apoptotic marker fraction (FRA). Initially, the sections were deparaffinized and rehydrated. Heat-induced antigen retrieval was then performed using 0.1 M citrate buffer (pH = 6) for 6 min. To block nonspecific binding sites, a 5% goat serum albumin (NGS) blocking buffer was applied, followed by permeabilization with a 1% Triton-X in PBS solution for 30 min. The anti-pCK primary antibody (mouse, IgG monoclonal AE1/AE3, Origene Technologies Inc.) and the anti-FRA primary antibody (rabbit, C-terminus, Millipore, Burlington, Massachusetts, USA) were applied at a 1:100 dilution and incubated overnight at 4 °C. Nuclear-specific dye Hoechst (Biotium, Hayward, CA, USA) was also applied (1:1000 dilution) to stain the nuclei of the epithelial cells blue. Subsequently, the secondary antimouse (goat IgG, Ex/Em: 490/525 nm, Biotium, Hayward, CA, USA) and antirabbit antibody (Biotium, Hayward, CA, USA cat. number 20033 goat antirabbit IgG 555) was used at a 1:200 dilution in a buffer containing 2% BSA in PBS for 1 h in the dark. Finally, the samples were mounted with a phenylamide- and glycerol-containing mounting medium, observed under a confocal laser microscope (Nikon EZ-C1, CLSM), and analyzed using the Ez-C1–3.20 software.

### Cell Studies

The embryonic mouse fibroblast cell line NIH3T3 was employed in the study to evaluate the toxicity of the developed MNs. The cells were cultured in Dulbecco’s modified Eagle’s medium (DMEM) supplemented with 10% fetal bovine serum (FBS), and 1% penicillin/streptomycin and incubated at 37 °C in a 5% v/v CO_2_ humidified atmosphere until 95% confluency.

### Cell Viability Study (MTT) Assay

Cell viability was assessed using the MTT assay after seeding (10^4^ cells/well) in 96-well plates. After 24 h of incubation, the cells were treated with the tested products. The protocol was adopted from the literature.^44,45^ Namely, leaching products of the printed MNs after 3 and 24 h and the plain coating film were tested to evaluate their toxicity on the cells. Upon 24 h of incubation, MTT solution (5 mg/mL) was added to the wells and left for another 4 h into the incubator. Consequently, the culture medium was removed, and DMSO was then added to the wells to dissolve the formazan crystals. The plate was then subjected to mild agitation for 15 min, and the absorbance of each well was measured using an ELISA plate reader at 540 nm.

### Preparation of 3D *In Vitro* Cell Model for Live/Dead Staining

A 3D model of the 3T3NIH cell line previously described in the literature^46^ was prepared by mixing a suspension of the cells (5 × 10^6^ cells/mL) in culture media with 2.8% w/v warm agarose solution at 1:1 volume ratio and placed onto a 6-well plate to solidify for 5 min. Cells in the agarose hydrogel were incubated for 24 h prior to MN application. The coated MNs were gently inserted into the hydrogel, and live/dead staining (Biotium, Fremont, California, USA) was performed after 24 h of treatment. Imaging of the 3D cell culture was followed using the Z-stack function of the confocal microscope (Zeiss LSM780, Zeiss, Oberkochen, Germany). The viability was further assessed by quantifying the live (green) and dead (red) signals at 3 different *Z*-positions.

### Data Analysis

All the results are presented as mean ± SD and all the experiments were conducted in triplicate. Student’s *t* test was performed to determine the level of significance (*p* ≤ 0.05).

## Results

### Characterization of the Coating Film

**Figure 1:**
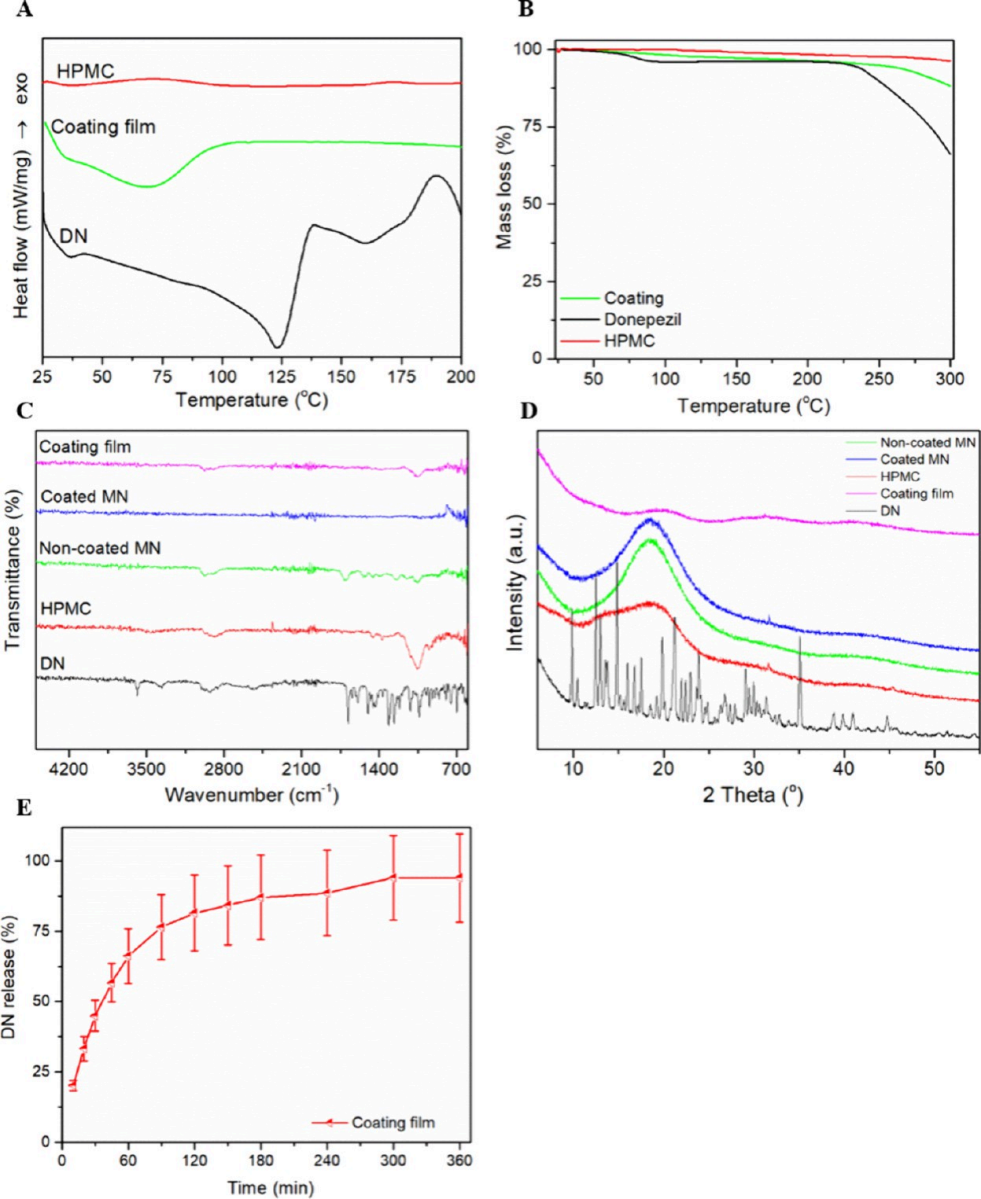
(A) DSC, (B) TGA, (C) FTIR, and (D) XRD analysis of the tested materials and the printed MNs. (E) *In vitro* release profile of DN from the coated MNs.

DSC, TGA, FTIR, and XRD analyses are presented in Figure 1. Thermal analysis was conducted for the API, the polymer, and the developed coating film to examine the temperature disparity existing between the sample and the raw materials. A sharp endothermic peak is observed at 123 °C for DN attributed to the melting point of the API.^47^ HPMC has a glass transition at 100 °C and the coating film presents a broad endotherm peak probably attributed to water and solvent residue evaporation at 60–90 °C. The characteristic endotherm peak of DN could not be identified in the DCS thermogram, indicating that the API is in the amorphous state inside the polymer matrix.^48^ TGA analysis (Figure 1B) showed no significant mass loss within the temperatures employed for the production of the MNs. This result confirmed that that the tested materials are stable and can be used for the production of the MN devices.

**Figure 2:**
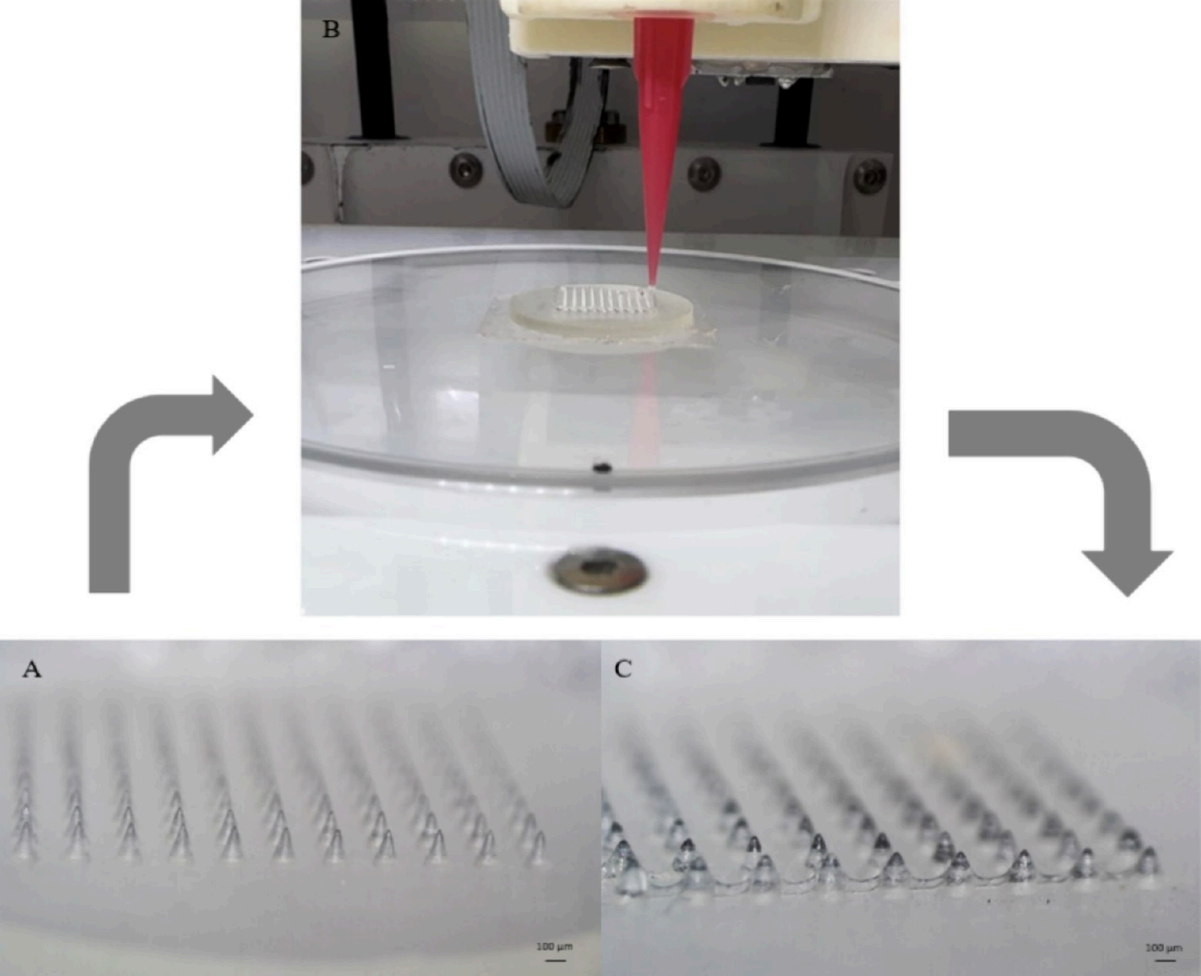
Microscopic pictures of (A) uncoated MNs and (C) coated MNs. (B) The coating process using an SSE.

FTIR analysis is presented in Figure 1C. All of the materials were evaluated with FTIR including the printed arrays (coated and uncoated ones) along with the raw materials. DN presents characteristic peaks at 1600 cm^–1^ and at 3500 cm^–1^ attributed to carbonyl and hydroxyl groups, respectively.^49^ These peaks are not present in the formulations suggesting that the API is molecularly dispersed inside the polymer matrix and is not simply attached to the film surface.^50^

XRD analysis (Figure 1D) confirmed the complete amorphization of the API onto the MNs and inside the coating film, as no characteristic peak of DN can be detected in the diffractogram of the other components. Overall, FTIR revealed the encapsulation of the drug inside the polymer matrix, while DSC and XRD confirmed the amorphization of DN after printing. Finally, TGA showed that all the materials are stable at the temperatures applied during the manufacturing process.

Figure 1E demonstrates the release of the API from the needles, indicating the sustained release profile of DN within 6 h. This study was conducted to ensure that the A rapid release is observed within the first hour and then the API is released in a slower rate. These results are in accordance with other dissolution studies conducted using HPMC as a carrier,^51^ and they confirm that the release of the API is mainly governed by the diffusion and erosion of the coating film.^52^ Moreover, the release data were optimally fitted to the Korsmeyer–Peppas model (*R*^2^ ≥ 0.9980), and DN exhibited an anomalous transport from the coating film to the release medium (*n* > 0.5).^48^

### Characterization of MN Arrays

#### Weight Uniformity

Weight uniformity was examined to evaluate the repeatability of both printing techniques to produce customized coated MNs with the same amount of coating and the same amount of API each time. The arrays were weighed before and after coating to ensure that the two processes are able to fabricate transdermal patches with the same features each time. The printed MNs were weighed before and after coating and their average weight and standard deviation were recorded. The mean values for the coated and uncoated MNs were found to be 0.506 g ± 3.54% and 0.499 g ± 3.67%, respectively. These limits are within the limits specified by USP.^53^ These results suggest that the two combined 3D printing processes have high accuracy and can produce transdermal drug delivery systems in a fast and reliable way.

### Optical Microscopy Studies

An optical microscope was used to visualize the MNs and measure their dimensions. Figure 2 depicts the MNs before and after coating with the SSE. Video S1 is also provided to visualize the coating procedure. The MNs were designed to be conical, 1 mm in length, and 0.5 mm in base diameter. Their dimensions were measured using ImageJ software, commonly used to measure MN dimensions.^2^ Four different arrays (coated and uncoated) were visualized from different angles to measure their length, base diameter, and tip diameter. For the uncoated arrays, their dimensions were 0.698 ± 0.06 mm in length, 0.550 ± 0.03 mm in base diameter, and 45 ± 14 μm in tip diameter. On the other hand, the coated MNs were 0.702 ± 0.06 mm in length, 0.630 ± 0.03 mm in base diameter, and 47 ± 16 μm in tip diameter. Overall, the dimensions are in good agreement with the digital design and the tips for both coated and noncoated MNs are sharp and capable of piercing.^54^ Coated MNs are slightly longer (*p* > 0.05) and have a bigger base diameter (*p* < 0.05) attributed to the deposition of the coating materials onto the MN. Actual needle length is lower than the digital design (1 mm compared to 0.702 and 0.698 mm in the printed arrays). This is due to the resin used for printing the arrays. After polymerization, and especially after curing, the printed objects tend to shrink due to the lower intramolecular distance of the cross-linked resin. Thus, the needles shrink after curing and finally they are shorter than the digital design.^1^ This was taken into account, and the digital design had intentionally bigger needles.

### SEM Studies

**Figure 3:**
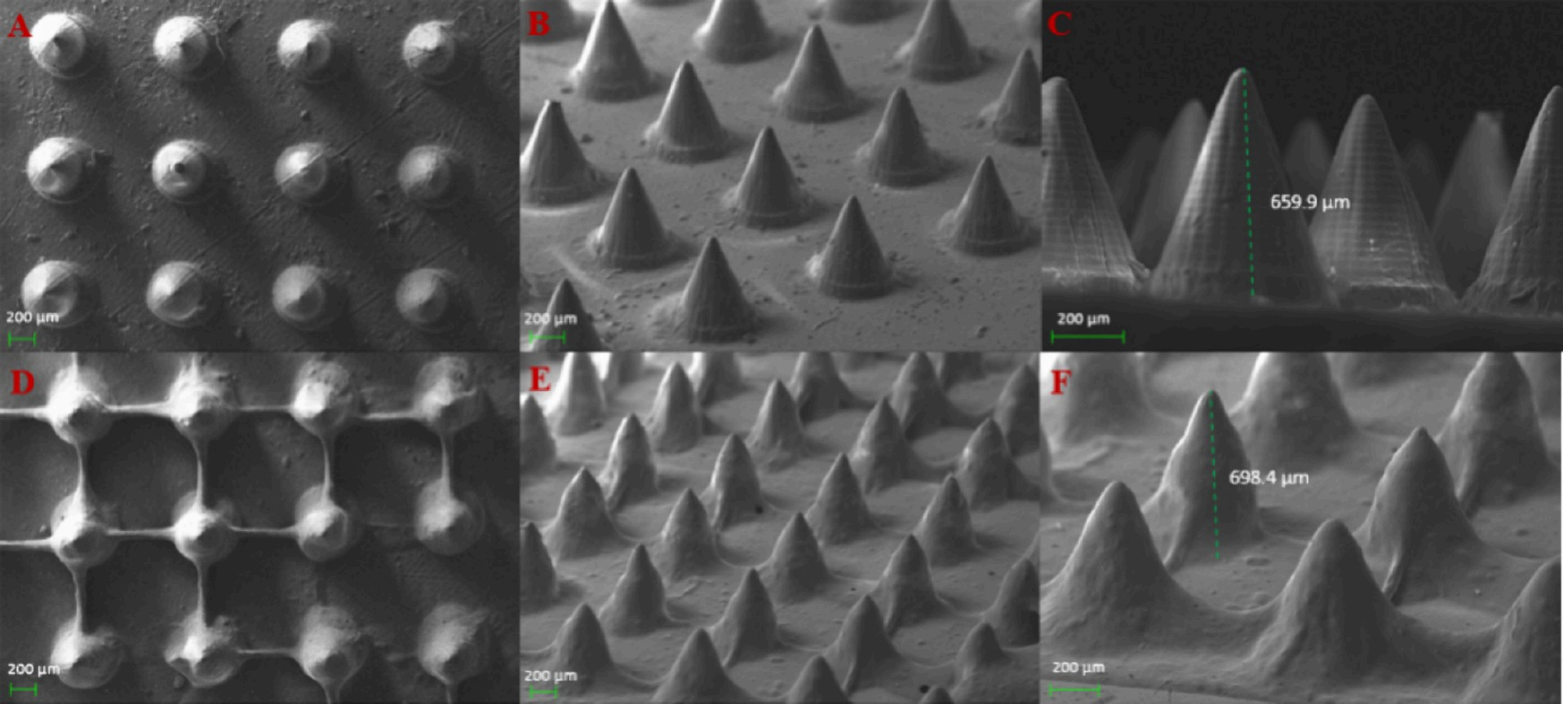
SEM images of (A–C) uncoated MN arrays and (D–F) coated MN arrays. SEM showed that the coating did not alter the shape of the needles and they did not collapse during the procedure. Most of the coating is deposited onto the MN tip, while some material is deposited onto the base due to leaking from the nozzle.

Figure 3 presents SEM images of the uncoated and coated MNs. SEM revealed that the needles did not deform or collapse during the coating process and the coating film simply deposited onto them. Their morphological features were also further examined. The arrays were printed directly onto the platform, and thus the layers are vertical to the surface. Most of the coating film was deposited onto the needles, but some material was also deposited onto the surface between the needles (Figure 3D). This is due to the travel moves of the printer and the leaking of the ink from the nozzle during the printing process that create these lines between the needles.

### Mechanical Properties and Insertion Test

**Figure 4:**
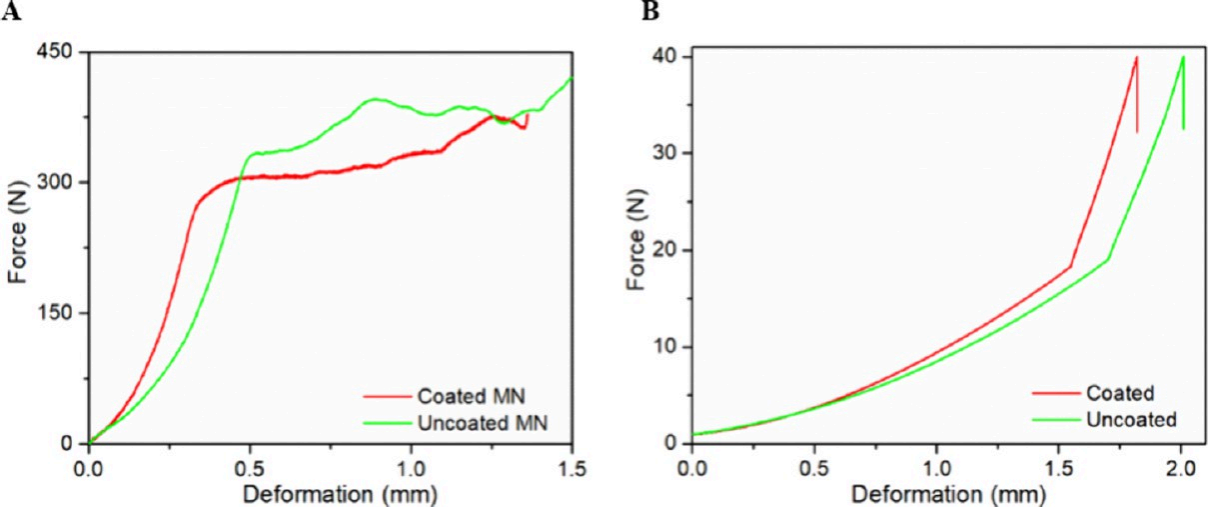
(A) Compression test and (B) insertion test of the coated and uncoated MN arrays. All the tests were conducted in triplicate and the presented graphs are the mean values of the MNs tested (0.006 < SD < 0.009).

A compression test was conducted to evaluate the ability of the MNs to pierce the skin without breaking or creating fragments that could lead to infection. The test was performed for both coated and uncoated MNs and the results are depicted in Figure 4A. The uncoated MNs can withstand forces up to 331 N while the coated ones are able to withstand 306 N. These forces are much higher than the forces applied during skin insertion, indicating that the developed arrays are safe for transdermal delivery.^44^ The coating process did not affect the mechanical properties of the needles, which is in accordance with other findings,^55^ suggesting that extrusion printing is just as effective as other means of coating MNs. The ability of the coated and uncoated MNs to perforate the human skin was examined during the insertion test using pig skin. The force–displacement curves of the MNs are shown in Figure 4B. The force applied for skin penetration was 40 N, a common force for MN application on the skin.^56^ As is evident from Figure 4B, the coated and uncoated MNs presented similar deformation values 1.81 and 2.01 mm, respectively. The penetration ability of the MNs is influenced by their tip diameter and by their length. It has been reported that needles shorter than 500 μm are not adequate for dependable identification of skin penetration. Moreover, one might suggest that a sharp MN tip is particularly crucial for penetrating real skin tissue because of the existence of the SC.^57^ Thus, the slight increase in deformation for the uncoated MNs is attributed to their sharper tips (45 μm compared to 47 μm for the coated ones).

### Delivery Efficiency of the Produced MN Arrays

It is important for the coated MNs to deliver effectively the coating into the skin, as the API is in the coating materials. Many coating materials have been introduced, namely, particles^58^ and polymers containing the drug.^59^ In the present study, a drug-loaded film was coated onto the needles by using extrusion printing. Artificial skin made from agarose was employed in this study to measure the efficiency of the needles to deliver the coating material effectively into the skin.^60^ The total amount of the drug deposited onto the MNs was also calculated to be 0.24 ± 0.07. The delivery efficiency for the printed MNs was 87 ± 6%. The amount of the drug that remained onto the array is attributed to the material deposited onto the base as described above, in the SEM analysis. After 5 min of insertion, the dissolution of the API is confirmed by quantifying the amount of the drug into the artificial skin.^59^

### *In Vitro* Permeation Study and Tape Stripping Assay

**Figure 5:**
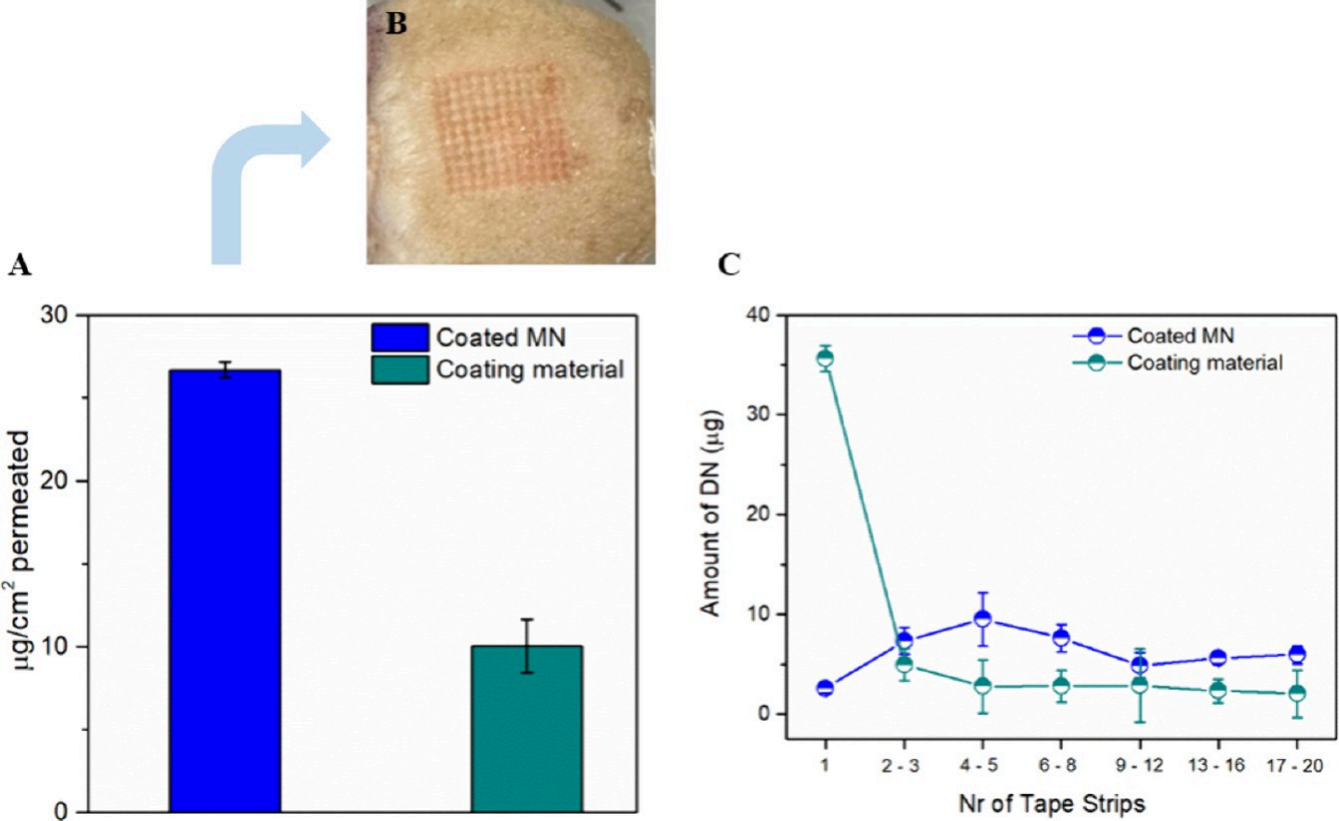
(A) Permeation study of DN using coated MNs and plain coating materials. (B) Perforation of the human skin samples with coated MNs. (C) Tape stripping of the SC after the application of MNs and coating material.

*In vitro* permeation study was conducted using full-thickness human skin to examine the ability of the MNs to increase the permeability of DN to the skin. The transport of DN was monitored for 24 h upon piercing with the coated MNs and application of the plain coating material. Figure 5A demonstrates the amount (μg/cm^2^) of DN permeated in each case. The MNs increased the permeation of DN up to 2.5-fold, which reveals that the printed MNs are a successful transdermal drug delivery system that can increase the absorption of the DN in a noninvasive way. The annular gap width was also calculated (4.62 ± 0.02 μm) using optical microscopy. The annular gap refers to the space around the needles through which the drug diffuses into the surrounding tissue, and it is basically governed by the needle size. Al-Qallaf and Das proposed a mathematical modeling and FEA simulations to optimize the dimensions of the MNs regarding the drug and the skin thickness highlighting the importance of the MN’s geometry and dimensions for a successful skin delivery.^61,62^ Coated MNs can increase significantly the amount of drug permeated due to the fact that they bypass the first skin barrier, the SC.^63^ Transdermal MN patches containing the API have been introduced for effective skin delivery. These patches are mainly composed of dissolving MNs.^17,64^ The results from the permeation study show that coated MNs are also effective and can enhance the absorption of DN. The coating process is easier, and the permeation profiles are comparable to the ones obtained from dissolving MNs. Coating the MNs with a polymer containing the drug resulted in an excellent permeation profile and in a controlled release of the API into the skin, as reported before in the literature.^65^Figure 5B presents the pattern of the coated MNs after piercing for 5 min of the human skin.

The results from the deposition of the API into the SC after tape stripping are presented in Figure 5C. It has been reported that the application of MNs can increase significantly the permeation of actives through the skin, as they can be visualized in the deepest layers.^66^ As expected, the application of the coating material led to the deposition of most drug onto the upper layer of the SC, whereas the application of the coated MNs led to increased deposition onto the deepest layers. Simple deposition of topical formulations shows that the penetration depth is decreased after removal of the SC, which is the case for the coating material, as the amount of DN declines in the innermost layers.^67,68^ However, the application of MNs has the opposite behavior as the activity is increased in tapes 4–5, confirming that the MNs have a significant impact in the penetration depth of the API. Moreover, the first layer of the SC has the smallest amount of DN, indicating that the printed MNs bypass successfully the first and most important layer of the skin. These results confirm that the produced MN device can significantly increase the permeation of DN from the skin.

### CLSM Studies

**Figure 6:**
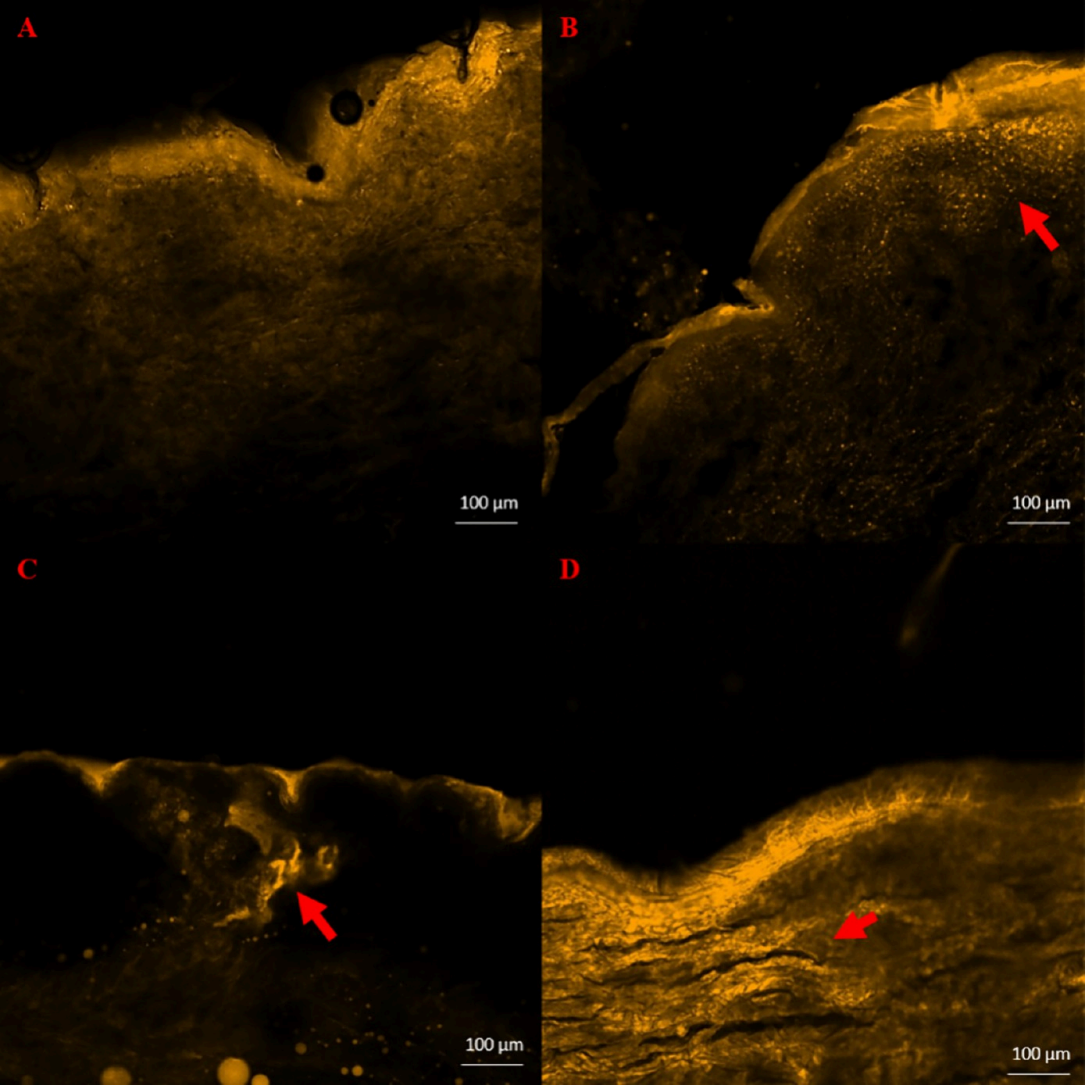
CLSM images depict the penetration and distribution of the model dye after piercing with the printed MNs (A) at 0 h, (B) at 4 h, (C) at 8 h, and (D) 24 h. Red arrows indicate the dye in the different skin layers.

CLSM has emerged as a well-established method for observing the distribution of drugs within the skin.^69−71^ The accumulation of the active in the SC, epidermis, and dermis can be visualized and the penetration depth can be determined. Video S2 depicts the coating procedure for the MNs with a Nile Red-loaded ink. Figure 6 depicts the penetration of Nile Red after 0, 4, 8, and 24 h of MN application. Fluorescence images were captured of vertical sections of the skin after MN treatment, and the penetration is indicated by red arrows. Within the first hours, the dye is mostly accumulated into the outermost layers of the skin while channels created by the MNs are visible in all images. In Figure 6C, the accumulation of the dye is mostly into the channels created by the MNs and the fluorescence signal is significantly lower on the surface. The channels facilitate the penetration of the active, and after 24 h, the dye has reached the dermis. Nile Red is a highly lipophilic substance and can permeate the skin using the transcellular pathway,^72^ and this is visible in Figure 6D, where the cells are stained red.

Other findings suggest that the MN application could enhance the penetration of lipophilic drugs for the management of Parkinson’s disease and CLSM was employed to further examine the channel formation into the skin that plays a key role in the drug absorption.^73^ CLSM confirmed that after MN penetration, the dye can reach the dermis and result in higher permeability values, which is also visible in Figure 5, and it is in good agreement with the literature.^74^ All these results suggest that the absorption of the drug could be increased and the release profile of API indicated that a sustained absorption is also possible, leading to lower frequency of administration and to better patient compliance.

### Histological Evaluation and Immunohistochemistry

**Figure 7:**
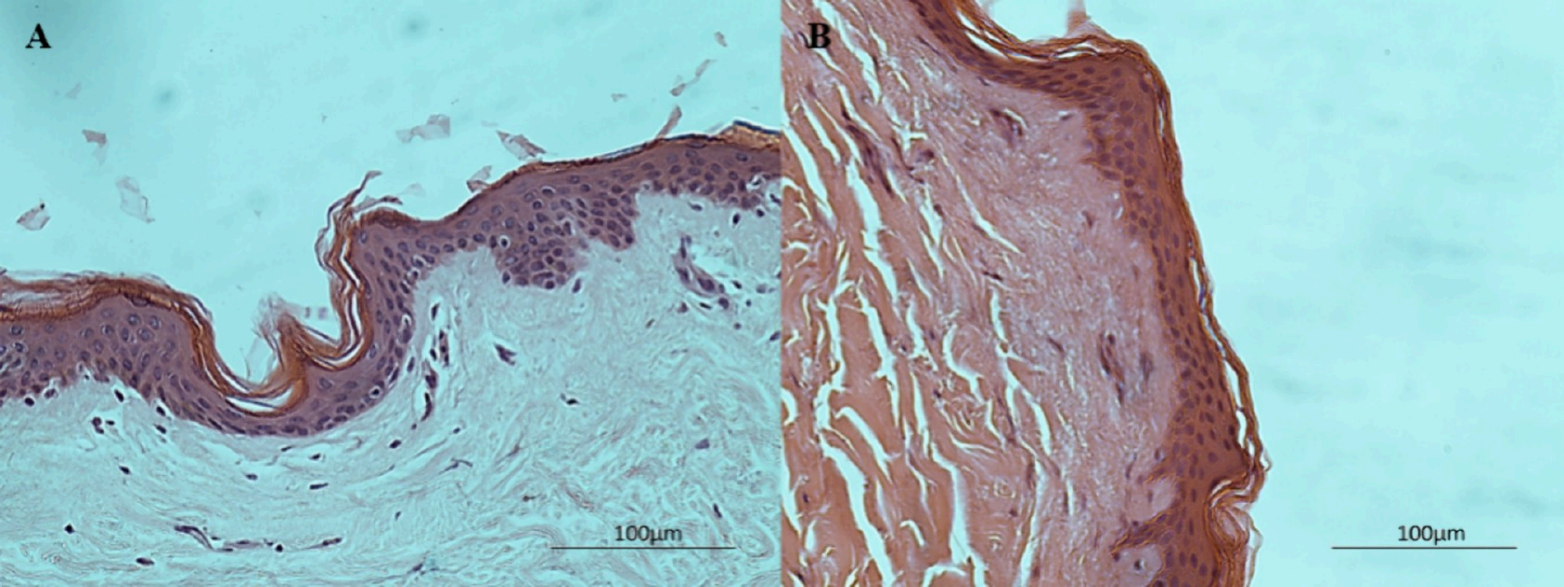
Histological evaluation of (A) control skin sample and (B) skin sample after piercing for 5 min with the MNs, using H&E staining. Use of MNs did not affected epidermal integrity neither its’ thickness.

Histological evaluation was conducted using H&E staining to ensure that the MNs are safe for transdermal applications. Figure 7 depicts microscopy images of the skin samples with and without MN treatment. The study showed that the MNs do not injure the epidermis, and the skin keeps its integrity after MN application. It has been reported that the skin can recover from MN penetration. Due to their small size and sharp geometry, MNs cause minimal skin damage, enabling quick recovery shortly after the application, and in this way, drug permeability is increased and the skin has no permanent damage.^75^

**Figure 8:**
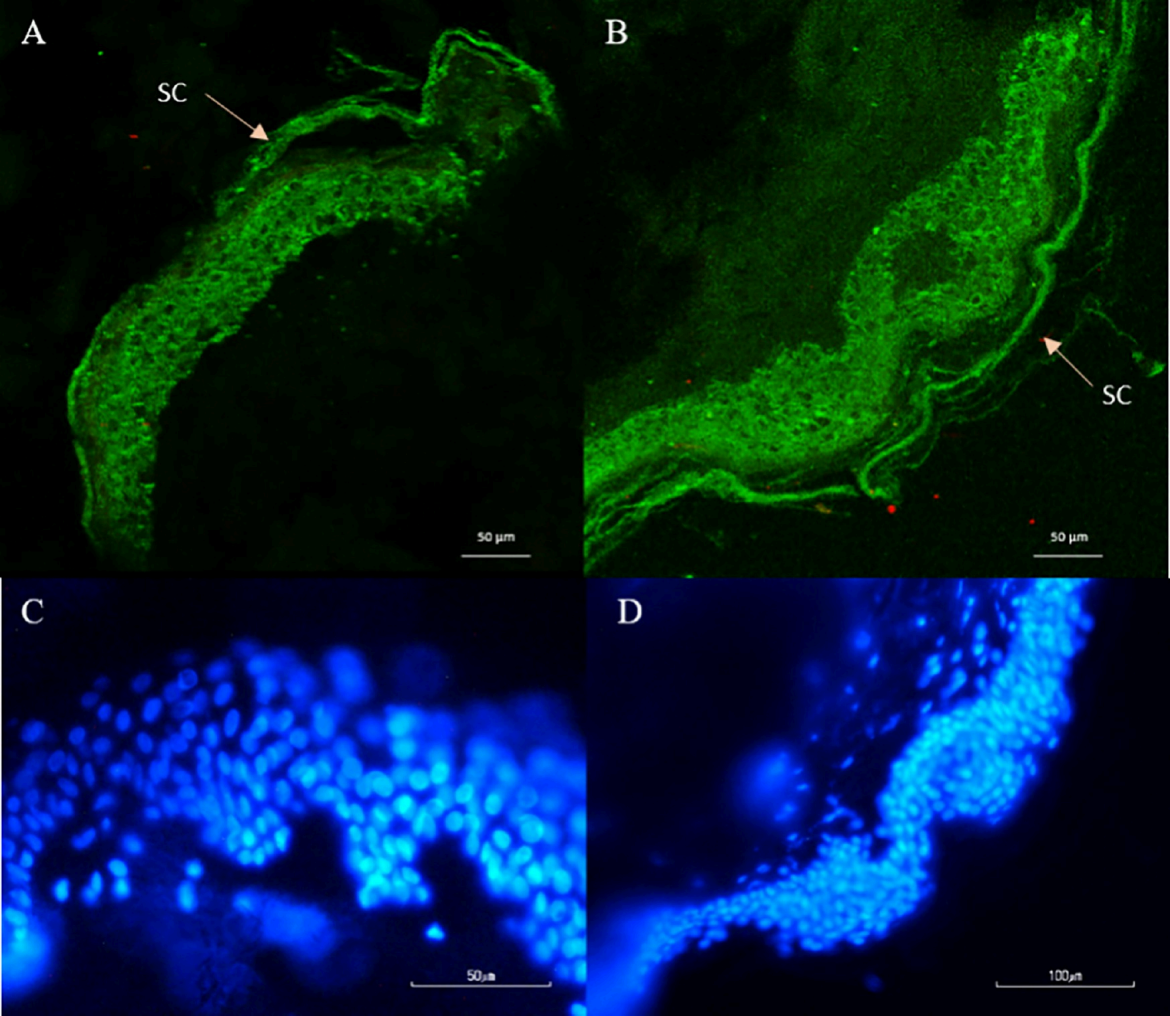
Representative images under fluorescence for pCK expression (green) and nuclei staining (blue) before MN application (A and C) and skin samples after MN application (B and D). The SC is indicated by white arrows. Immunofluorescence studies confirmed H&E staining by showing the integrity of the epidermis after the MNs application.

An immunohistochemical study was also conducted to confirm that the produced MNs are completely safe for on-body applications. The epithelial cells strongly express pCK and it is often used as a marker to label this cell population.^76^ To further examine whether the produced MNs trigger any apoptotic pathway, FRA staining was also conducted to reveal any cells that are undergoing DNA fragmentation.^77^Figure 8 shows the images under fluorescence, highlighting the epithelial cells of the epidermis in green (pCK) and the apoptotic cells in red (FRA). No red cells were observed in the tissue samples, indicating that there was no toxic effect after MN treatment. These results are in good agreement with the literature, as the size of the needle is too small and it cannot cause any damage to the tissues. Moreover, the dermal thickness is not affected, which is a good indicator that the needles will not trigger any skin diseases related to this.^78^

### Cell Studies

#### MTT-Assay

**Figure 9:**
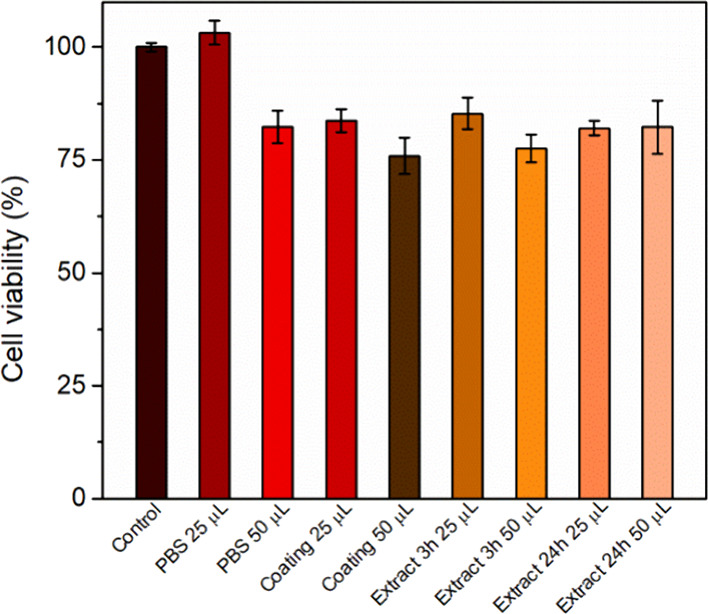
Cell viability study using the MTT assay after 24 h incubation of NIH3T3 cells with the coating material and with printed resin extracts.

To ensure the safety of the produced MNs, a cell viability study was conducted. The commercial resin used for the production of these arrays is considered safe as it is classified as biocompatible, and it has been reported in the literature for the production of different MN applications.^1,79^ Both the extracts and the HPMC coating were tested, and the results are presented in Figure 9. Different concentrations of the tested samples were also examined to determine the concentration-dependent cytotoxicity. Untreated and PBS-treated cells are also presented as control groups. None of the tested concentrations seem to decrease the cell viability (%) as all samples had over 77% viability. As the viability remained over 77% in all cases, the tested samples are considered safe and nontoxic.^80^ The assay was conducted only after 24 h of incubation, as the printed MNs penetrated the skin only for 5 min and the coating material released the drug within 6 h. Thus, in these timeframes, the materials used are considered safe for transdermal application.

### Live/Dead Staining of the 3D *In Vitro* Cell Model

**Figure 10:**
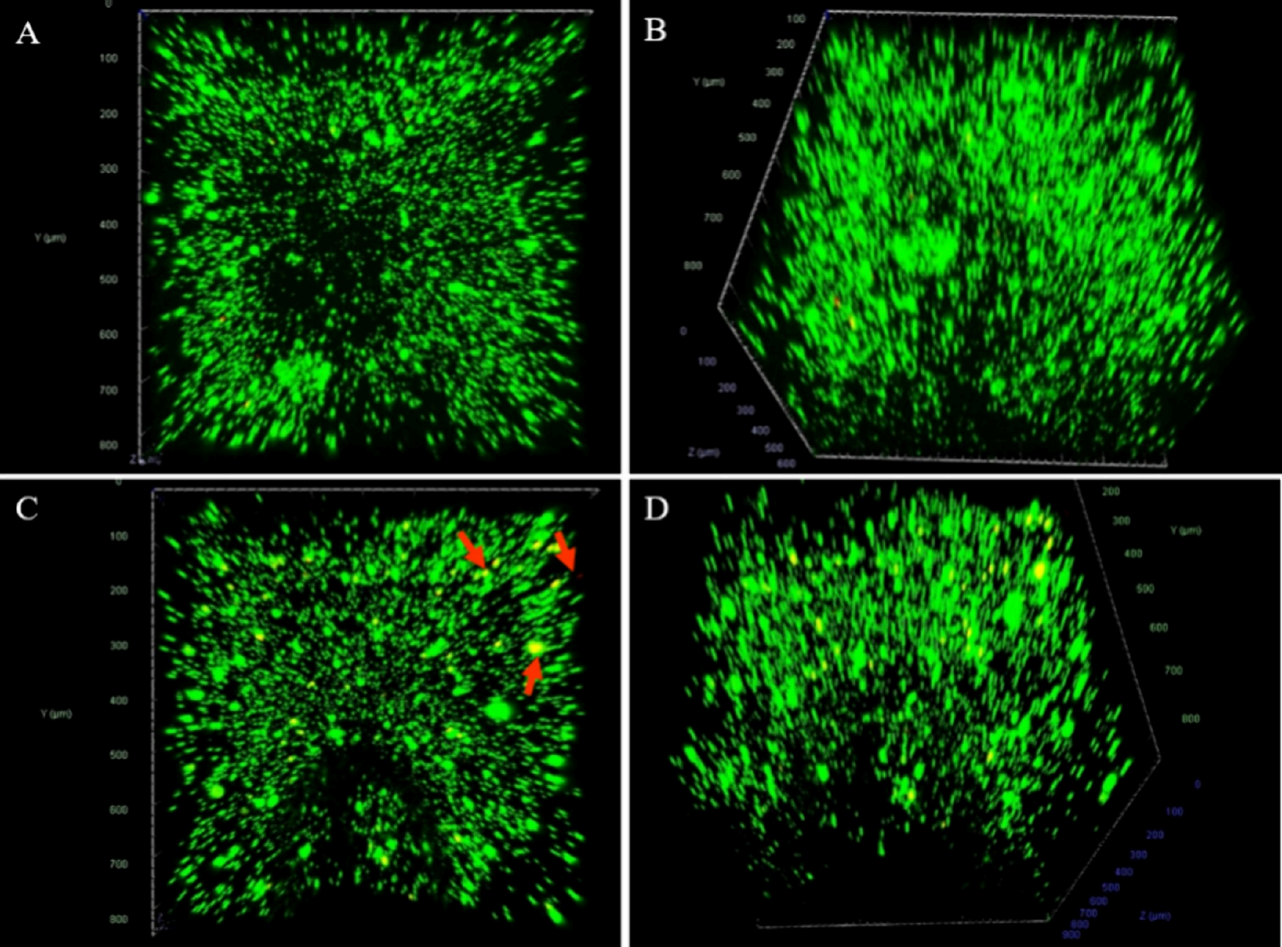
Live/dead staining of the 3D *in vitro* model cell. Live cells are stained green, and dead cells are stained red. (A) 2D and (B) 3D images of the agarose cell culture after no treatment (control group). (C) 2D and (D) 3D images were obtained after 24 h of treatment with the printed MNs.

To further examine the effect of the MN penetration onto the cells, a 3D cell model was developed and the MN was gently inserted into them. After 24 h, the cell model was subjected to live/dead staining and the fluorescence images are displayed in Figure 10. No significant changes are observed before and after MN penetration. In Figure 10C,D, some dead cells are located on the surface of the culture (indicated by the red arrows), which is probably attributed to the mechanical force used to pierce the agarose gel. This is a normal effect as the culture is exposed to the needles directly as there are no keratinocytes to protect them. These tests were conducted to ensure the nontoxic profile of the printed MNs. The resins used to print the arrays are composed of monomers that are usually toxic, and they might irritate the skin during application. However, after cross-linking, they are considered safe and nontoxic. The cross-linking of the monomers is most important as it determines the mechanical properties of the objects and also may affect the cell viability.^81^ To avoid these problems, the printed MNs were fully cross-linked into the UV chamber prior to coating, and inally the produced arrays had no toxic effect in the cells.

## Conclusions

In the present study, two different 3D printing processes were combined to develop coated MNs for the transdermal delivery of DN. The two processes can produce in a fast and reliable way coated arrays with the appropriate amount of activity each time. The coating film was evaluated with regard to its physiochemical properties. The XRD study confirmed the amorphization of the API onto the MN and the release study showed that DN is fully released from the MN within 6 h. The weight uniformity suggested that mass production is feasible, and microscopy and SEM studies revealed that the printed MNs are appropriate for piercing the skin. Mechanical and insertion tests indicated that the needles can effectively pierce the skin without breaking or creating fragments that could irritate or infect the skin. Delivery efficiency and permeation studies were further conducted to evaluate the ability of the coating material to deliver API across the skin. The results proved that the MNs can increase the permeation of the API up to 2.5-fold, only after 5 min of piercing. The distribution of the drug was examined using CLSM, where the API was substituted with Nile Red, a model dye. Nile Red reached the dermis after 24 h, while the transcellular route of transportation was visualized. These results are promising as they reveal a sustained release for the API and in this way, the frequency of administration can be decreased. Finally, immunocytochemistry studies and cell studies using NIH3T3 fibroblasts demonstrated that the printed MNs are safe for transdermal applications and have no cytotoxic effects. Overall, combining two additive manufacturing processes resulted in the production of customized drug delivery systems for the transdermal delivery of actives that are highly potent and can solve the problem of personalization for each patient.

## Supporting Information Available

Video S1: Coating the MNs with DN-loaded ink (MP4)
Video S2: Coating the MNs with Nile red-loaded ink (MP4)

## References

[ref1] Monou P. K.; Andriotis E. G.; Tsongas K.; Tzimtzimis E. K.; Katsamenis O. L.; Tzetzis D.; Anastasiadou P.; Ritzoulis C.; Vizirianakis I. S.; Andreadis D.; Fatouros D. G. Fabrication of 3D Printed Hollow Microneedles by Digital Light Processing for the Buccal Delivery of Actives. ACS Biomater Sci. Eng. 2023, 9 (8), 5072–5083. 10.1021/acsbiomaterials.3c00116. 37528336

[ref2] El-Sayed N.; Vaut L.; Schneider M. Customized Fast-Separable Microneedles Prepared with the Aid of 3D Printing for Nanoparticle Delivery. Eur. J. Pharm. Biopharm. 2020, 154, 166–174. 10.1016/j.ejpb.2020.07.005. 32659323

[ref3] Wu M.; Zhang Y.; Huang H.; Li J.; Liu H.; Guo Z.; Xue L.; Liu S.; Lei Y. Assisted 3D Printing of Microneedle Patches for Minimally Invasive Glucose Control in Diabetes. Materials Science and Engineering: C 2020, 117, 111299 10.1016/j.msec.2020.111299. 32919660

[ref4] Economidou S. N.; Pere C. P. P.; Reid A.; Uddin Md. J.; Windmill J. F. C.; Lamprou D. A.; Douroumis D. 3D Printed Microneedle Patches Using Stereolithography (SLA) for Intradermal Insulin Delivery. Materials Science and Engineering: C 2019, 102, 743–755. 10.1016/j.msec.2019.04.063. 31147046

[ref5] Mathew E.; Pitzanti G.; Gomes dos Santos A. L.; Lamprou D. A. Optimization of Printing Parameters for Digital Light Processing 3D Printing of Hollow Microneedle Arrays. Pharmaceutics 2021, 13 (11), 1837 10.3390/pharmaceutics13111837. 34834250 PMC8622592

[ref6] Chatzitaki A.-T.; Eleftheriadis G.; Tsongas K.; Tzetzis D.; Spyros A.; Vizirianakis I. S.; Fatouros D. G. Fabrication of 3D-Printed Octreotide Acetate-Loaded Oral Solid Dosage Forms by Means of Semi-Solid Extrusion Printing. Int. J. Pharm. 2023, 632, 122569 10.1016/j.ijpharm.2022.122569. 36592893

[ref7] Takashima H.; Tagami T.; Kato S.; Pae H.; Ozeki T.; Shibuya Y. Three-Dimensional Printing of an Apigenin-Loaded Mucoadhesive Film for Tailored Therapy to Oral Leukoplakia and the Chemopreventive Effect on a Rat Model of Oral Carcinogenesis. Pharmaceutics 2022, 14 (8), 1575 10.3390/pharmaceutics14081575. 36015201 PMC9415331

[ref8] Alzheimer’s Disease International; Patterson C. World Alzheimer Report 2018: The State of the Art of Dementia Research: New Frontiers, Alzheimer’s Disease International 2018.

[ref9] Hussain R.; Khan S.; Ullah H.; Ali F.; Khan Y.; Sardar A.; Iqbal R.; Ataya F. S.; El-Sabbagh N. M.; Batiha G. E.-S. Benzimidazole-Based Schiff Base Hybrid Scaffolds: A Promising Approach to Develop Multi-Target Drugs for Alzheimer’s Disease. Pharmaceuticals (Basel) 2023, 16 (9), 1278 10.3390/ph16091278. 37765088 PMC10535318

[ref10] Abraham J. T.; Maharifa H. N. S.; Hemalatha S. In Silico Molecular Docking Approach Against Enzymes Causing Alzheimer’s Disease Using Borassus Flabellifer Linn. Appl. Biochem. Biotechnol. 2022, 194 (4), 1804–1813. 10.1007/s12010-021-03779-3. 35013923 PMC8747846

[ref11] Mahajan E.; Raja A.; Sharma A. raj; Jain A.; K. Prabha P.; Prakash A.; Medhi B. To Evaluate the Effect of Endothelin Receptor Agonist IRL-1620 Alone and in Combination with Donepezil in Modulating Neurodegeneration Elicited by Amyloid-β in Rats. Exp. Neurol. 2024, 375, 114720 10.1016/j.expneurol.2024.114720. 38342181

[ref12] Chen R.; Chan P.-T.; Chu H.; Lin Y.-C.; Chang P.-C.; Chen C.-Y.; Chou K.-R. Treatment Effects between Monotherapy of Donepezil versus Combination with Memantine for Alzheimer Disease: A Meta-Analysis. PLoS One 2017, 12 (8), e0183586 10.1371/journal.pone.0183586. 28827830 PMC5565113

[ref13] Handa M.; Sanap S. N.; Bhatta R. S.; Patil G. P.; Ghose S.; Singh D. P.; Shukla R. Combining Donepezil and Memantine via Mannosylated PLGA Nanoparticles for Intranasal Delivery: Characterization and Preclinical Studies. Biomaterials Advances 2023, 154, 213663 10.1016/j.bioadv.2023.213663. 37865027

[ref14] Farlow M.; Veloso F.; Moline M.; Yardley J.; Brand-Schieber E.; Bibbiani F.; Zou H.; Hsu T.; Satlin A. Safety and Tolerability of Donepezil 23 Mg in Moderate to Severe Alzheimer’s Disease. BMC Neurol 2011, 11, 57 10.1186/1471-2377-11-57. 21612646 PMC3126705

[ref15] Ertas B.; Onay I. N.; Yilmaz-Goler A. M.; Karademir-Yilmaz B.; Aslan I.; Cam M. E. A Novel High-Efficiency Transdermal Patches for Combinational Therapy of Alzheimer’s Disease: Donepezil/Vitamin B12-Loaded Nanofibers. Journal of Drug Delivery Science and Technology 2023, 89, 104963 10.1016/j.jddst.2023.104963.

[ref16] Mendes I. T.; Ruela A. L. M.; Carvalho F. C.; Freitas J. T. J.; Bonfilio R.; Pereira G. R. Development and Characterization of Nanostructured Lipid Carrier-Based Gels for the Transdermal Delivery of Donepezil. Colloids Surf., B 2019, 177, 274–281. 10.1016/j.colsurfb.2019.02.007. 30763792

[ref17] Kim J.-Y.; Han M.-R.; Kim Y.-H.; Shin S.-W.; Nam S.-Y.; Park J.-H. Tip-Loaded Dissolving Microneedles for Transdermal Delivery of Donepezil Hydrochloride for Treatment of Alzheimer’s Disease. Eur. J. Pharm. Biopharm. 2016, 105, 148–155. 10.1016/j.ejpb.2016.06.006. 27288938

[ref18] Kearney M.-C.; Caffarel-Salvador E.; Fallows S. J.; McCarthy H. O.; Donnelly R. F. Microneedle-Mediated Delivery of Donepezil: Potential for Improved Treatment Options in Alzheimer’s Disease. Eur. J. Pharm. Biopharm. 2016, 103, 43–50. 10.1016/j.ejpb.2016.03.026. 27018330

[ref19] Anjani Q. K.; Cárcamo-Martínez Á.; Wardoyo L. A. H.; Moreno-Castellanos N.; Sabri A. H. B.; Larrañeta E.; Donnelly R. F. MAP-Box: A Novel, Low-Cost and Easy-to-Fabricate 3D-Printed Box for the Storage and Transportation of Dissolving Microneedle Array Patches. Drug Delivery and Transl. Res. 2024, 14 (1), 208–222. 10.1007/s13346-023-01393-w. PMC10746783 37477867

[ref20] McKenna P. E.; Abbate M. T. A.; Vora L. K.; Sabri A. H.; Peng K.; Volpe-Zanutto F.; Tekko I. A.; Permana A. D.; Maguire C.; Dineen D.; Kearney M.-C.; Larrañeta E.; Paredes A. J.; Donnelly R. F. Polymeric Microarray Patches for Enhanced Transdermal Delivery of the Poorly Soluble Drug Olanzapine. ACS Appl. Mater. Interfaces 2023, 15 (26), 31300–31319. 10.1021/acsami.3c05553. 37349320 PMC10326804

[ref21] Sozio P.; Cerasa L. S.; Marinelli L.; Di Stefano A. Transdermal Donepezil on the Treatment of Alzheimer’s Disease. Neuropsychiatr Dis Treat 2012, 8, 361–368. 10.2147/NDT.S16089. 22942647 PMC3428243

[ref22] Kirkby M.; Hutton A. R. J.; Donnelly R. F. Microneedle Mediated Transdermal Delivery of Protein, Peptide and Antibody Based Therapeutics: Current Status and Future Considerations. Pharm. Res. 2020, 37 (6), 117 10.1007/s11095-020-02844-6. 32488611 PMC7266419

[ref23] Economidou S. N.; Lamprou D. A.; Douroumis D. 3D Printing Applications for Transdermal Drug Delivery. Int. J. Pharm. 2018, 544 (2), 415–424. 10.1016/j.ijpharm.2018.01.031. 29355656

[ref24] Guillot A. J.; Martínez-Navarrete M.; Garrigues T. M.; Melero A. Skin Drug Delivery Using Lipid Vesicles: A Starting Guideline for Their Development. J. Controlled Release 2023, 355, 624–654. 10.1016/j.jconrel.2023.02.006. 36775245

[ref25] Prausnitz M. R.; Langer R. Transdermal Drug Delivery. Nat. Biotechnol. 2008, 26 (11), 1261–1268. 10.1038/nbt.1504. 18997767 PMC2700785

[ref26] Paredes A. J.; Ramöller I. K.; McKenna P. E.; Abbate M. T. A.; Volpe-Zanutto F.; Vora L. K.; Kilbourne-Brook M.; Jarrahian C.; Moffatt K.; Zhang C.; Tekko I. A.; Donnelly R. F. Microarray Patches: Breaking down the Barriers to Contraceptive Care and HIV Prevention for Women across the Globe. Adv. Drug Delivery Rev. 2021, 173, 331–348. 10.1016/j.addr.2021.04.002. 33831475

[ref27] Donnelly R. F.; McCrudden M. T. C.; Zaid Alkilani A.; Larrañeta E.; McAlister E.; Courtenay A. J.; Kearney M.-C.; Singh T. R. R.; McCarthy H. O.; Kett V. L.; Caffarel-Salvador E.; Al-Zahrani S.; Woolfson A. D. Hydrogel-Forming Microneedles Prepared from “Super Swelling” Polymers Combined with Lyophilised Wafers for Transdermal Drug Delivery. PLoS One 2014, 9 (10), e111547 10.1371/journal.pone.0111547. 25360806 PMC4216095

[ref28] Kim Y.-C.; Park J.-H.; Prausnitz M. R. Microneedles for Drug and Vaccine Delivery. Adv. Drug Delivery Rev. 2012, 64 (14), 1547–1568. 10.1016/j.addr.2012.04.005. PMC3419303 22575858

[ref29] Hirakawa Y.; Ueda H.; Miyano T.; Kamiya N.; Goto M. New Insight into Transdermal Drug Delivery with Supersaturated Formulation Based on Co-Amorphous System. Int. J. Pharm. 2019, 569, 118582 10.1016/j.ijpharm.2019.118582. 31381987

[ref30] Stott P. W.; Williams A. C.; Barry B. W. Transdermal delivery from eutectic systems: enhanced permeation of a model drug, ibuprofen. J. Controlled Release 1998, 50 (1), 297–308. 10.1016/S0168-3659(97)00153-3. 9685897

[ref31] Andrews S.; Lee J. W.; Choi S.-O.; Prausnitz M. R. Transdermal Insulin Delivery Using Microdermabrasion. Pharm. Res. 2011, 28 (9), 2110–2118. 10.1007/s11095-011-0435-4. 21499837 PMC3152630

[ref32] Tsai J.-C.; Guy R. H.; Thornfeldt C. R.; Gao W. N.; Feingold K. R.; Elias P. M. Metabolic Approaches To Enhance Transdermal Drug Delivery. 1. Effect of Lipid Synthesis Inhibitors. J. Pharm. Sci. 1996, 85 (6), 643–648. 10.1021/js950219p. 8773963

[ref33] Verbaan F. J.; Bal S. M.; van den Berg D. J.; Groenink W. H. H.; Verpoorten H.; Lüttge R.; Bouwstra J. A. Assembled Microneedle Arrays Enhance the Transport of Compounds Varying over a Large Range of Molecular Weight across Human Dermatomed Skin. J. Controlled Release 2007, 117 (2), 238–245. 10.1016/j.jconrel.2006.11.009. 17196697

[ref34] Verbaan F. J.; Bal S. M.; van den Berg D. J.; Dijksman J. A.; van Hecke M.; Verpoorten H.; van den Berg A.; Luttge R.; Bouwstra J. A. Improved Piercing of Microneedle Arrays in Dermatomed Human Skin by an Impact Insertion Method. J. Controlled Release 2008, 128 (1), 80–88. 10.1016/j.jconrel.2008.02.009. 18394741

[ref35] Bal S. M.; Caussin J.; Pavel S.; Bouwstra J. A. In Vivo Assessment of Safety of Microneedle Arrays in Human Skin. Eur. J. Pharm. Sci. 2008, 35 (3), 193–202. 10.1016/j.ejps.2008.06.016. 18657610

[ref36] van der Maaden K.; Jiskoot W.; Bouwstra J. Microneedle Technologies for (Trans)Dermal Drug and Vaccine Delivery. J. Controlled Release 2012, 161 (2), 645–655. 10.1016/j.jconrel.2012.01.042. 22342643

[ref37] Anjani Q. K.; Sabri A. H. B.; Utomo E.; Domínguez-Robles J.; Donnelly R. F. Elucidating the Impact of Surfactants on the Performance of Dissolving Microneedle Array Patches. Mol. Pharmaceutics 2022, 19 (4), 1191–1208. 10.1021/acs.molpharmaceut.1c00988. PMC9097526 35235330

[ref38] Fitri A. M. N.; Mahfufah U.; Aziz S. B. A.; Sultan N. A. F.; Mahfud M. A. S.; Saputra M. D.; Elim D.; Bakri N. F.; Arjuna A.; Sari Y. W.; Domínguez-Robles J.; Pamornpathomkul B.; Mir M.; Permana A. D. Enhancement of Skin Localization of β-Carotene from Red Fruit (*Pandanus Conoideus* Lam.) Using Solid Dispersion-Thermoresponsive Gel Delivered via Polymeric Solid Microneedles. Int. J. Pharm. 2024, 660, 124307 10.1016/j.ijpharm.2024.124307. 38852748

[ref39] Lv J.; Zhao J.; Li X.; Ling G.; Zhang P. Preparation of a Novel Hyaluronic Acid-Based Separable Hydrogel Microneedle with Niacinamide to Treat Pigment Deposition Using Solvent-Free Solid-State Crosslinking Method. Eur. Polym. J. 2024, 210, 113003 10.1016/j.eurpolymj.2024.113003.

[ref40] Bhadale R. S.; Londhe V. Y. Solid Microneedle Assisted Transepidermal Delivery of Iloperidone Loaded Film: Characterization and Skin Deposition Studies. Journal of Drug Delivery Science and Technology 2023, 79, 104028 10.1016/j.jddst.2022.104028.

[ref41] Chandbadshah S. B. V. J.; Mannayee G. Structural Analysis and Simulation of Solid Microneedle Array for Vaccine Delivery Applications. Mater. Today: Proc. 2022, 65, 3774–3779. 10.1016/j.matpr.2022.06.483. 35855948 PMC9277466

[ref42] Le Z.; Yu J.; Quek Y. J.; Bai B.; Li X.; Shou Y.; Myint B.; Xu C.; Tay A. Design Principles of Microneedles for Drug Delivery and Sampling Applications. Mater. Today 2023, 63, 137–169. 10.1016/j.mattod.2022.10.025.

[ref43] Yao W.; Li D.; Zhao Y.; Zhan Z.; Jin G.; Liang H.; Yang R. 3D Printed Multi-Functional Hydrogel Microneedles Based on High-Precision Digital Light Processing. Micromachines 2020, 11 (1), 17 10.3390/mi11010017. PMC7019295 31877987

[ref44] Xenikakis I.; Tsongas K.; Tzimtzimis E. K.; Zacharis C. K.; Theodoroula N.; Kalogianni E. P.; Demiri E.; Vizirianakis I. S.; Tzetzis D.; Fatouros D. G. Fabrication of Hollow Microneedles Using Liquid Crystal Display (LCD) Vat Polymerization 3D Printing Technology for Transdermal Macromolecular Delivery. Int. J. Pharm. 2021, 597, 120303 10.1016/j.ijpharm.2021.120303. 33540009

[ref45] Guerrero-Gironés J.; López-García S.; Pecci-Lloret M. R.; Pecci-Lloret M. P.; Rodríguez Lozano F. J.; García-Bernal D. *In Vitro* Biocompatibility Testing of 3D Printing and Conventional Resins for Occlusal Devices. Journal of Dentistry 2022, 123, 104163 10.1016/j.jdent.2022.104163. 35577252

[ref46] Ning X.; Wiraja C.; Chew W. T. S.; Fan C.; Xu C. Transdermal Delivery of Chinese Herbal Medicine Extract Using Dissolvable Microneedles for Hypertrophic Scar Treatment. Acta Pharmaceutica Sinica B 2021, 11 (9), 2937–2944. 10.1016/j.apsb.2021.03.016. 34589406 PMC8463281

[ref47] Quan P.; Guo W.; LinYang; Cun D.; Yang M. Donepezil Accelerates the Release of PLGA Microparticles via Catalyzing the Polymer Degradation Regardless of the End Groups and Molecular Weights. Int. J. Pharm. 2023, 632, 122566 10.1016/j.ijpharm.2022.122566. 36586633

[ref48] Monou P. K.; Andriotis E. G.; Bouropoulos N.; Panteris E.; Akrivou M.; Vizirianakis I. S.; Ahmad Z.; Fatouros D. G. Engineered Mucoadhesive Microparticles of Formoterol/Budesonide for Pulmonary Administration. European Journal of Pharmaceutical Sciences 2021, 165, 105955 10.1016/j.ejps.2021.105955. 34298141

[ref49] Poormoghadam D.; Ghollasi M.; Babavalian H.; Tabasi A.; Shams M.; Goodarzi V.; Salimi A. Modification and Characterization of an Innovative Polyvinyl Alcohol-45S5 Bioactive Glass Nanocomposite Scaffold Containing Donepezil Hydrochloride for Bone Tissue Engineering Applications. Mater. Lett. 2021, 300, 130160 10.1016/j.matlet.2021.130160.

[ref50] Mavrokefalou E.; Monou P. K.; Tzetzis D.; Bouropoulos N.; Vizirianakis I. S.; Fatouros D. G. Preparation and *in Vitro* Evaluation of Electrospun Sodium Alginate Fiber Films for Wound Healing Applications. Journal of Drug Delivery Science and Technology 2023, 81, 104298 10.1016/j.jddst.2023.104298.

[ref51] Cheng Y.; Qin H.; Acevedo N. C.; Jiang X.; Shi X. 3D Printing of Extended-Release Tablets of Theophylline Using Hydroxypropyl Methylcellulose (HPMC) Hydrogels. Int. J. Pharm. 2020, 591, 119983 10.1016/j.ijpharm.2020.119983. 33065220

[ref52] Vanhoorne V.; Janssens L.; Vercruysse J.; De Beer T.; Remon J. P.; Vervaet C. Continuous Twin Screw Granulation of Controlled Release Formulations with Various HPMC Grades. Int. J. Pharm. 2016, 511 (2), 1048–1057. 10.1016/j.ijpharm.2016.08.020. 27521702

[ref53] Ganatra P.; Jyothish L.; Mahankal V.; Sawant T.; Dandekar P.; Jain R. Drug-Loaded Vegan Gummies for Personalized Dosing of Simethicone: A Feasibility Study of Semi-Solid Extrusion-Based 3D Printing of Pectin-Based Low-Calorie Drug Gummies. Int. J. Pharm. 2024, 651, 123777 10.1016/j.ijpharm.2024.123777. 38181992

[ref54] Kawre S.; Suryavanshi P.; Lalchandani D. S.; Deka M. K.; Kumar Porwal P.; Kaity S.; Roy S.; Banerjee S. Bioinspired Labrum-Shaped Stereolithography (SLA) Assisted 3D Printed Hollow Microneedles (HMNs) for Effectual Delivery of Ceftriaxone Sodium. Eur. Polym. J. 2024, 204, 112702 10.1016/j.eurpolymj.2023.112702.

[ref55] Jakka D.; Matadh A. V.; Shankar V. K.; Shivakumar H. N.; Narasimha Murthy S. Polymer Coated Polymeric (PCP) Microneedles for Controlled Delivery of Drugs (Dermal and Intravitreal). J. Pharm. Sci. 2022, 111 (10), 2867–2878. 10.1016/j.xphs.2022.05.023. 35662543 PMC10775835

[ref56] Ali R.; Mehta P.; Kyriaki Monou P.; Arshad M. S.; Panteris E.; Rasekh M.; Singh N.; Qutachi O.; Wilson P.; Tzetzis D.; Chang M.-W.; Fatouros D. G.; Ahmad Z. Electrospinning/Electrospraying Coatings for Metal Microneedles: A Design of Experiments (DOE) and Quality by Design (QbD) Approach. Eur. J. Pharm. Biopharm. 2020, 156, 20–39. 10.1016/j.ejpb.2020.08.023. 32871196

[ref57] Bisgaard S. I.; Nguyen L. Q.; Bøgh K. L.; Keller S. S. Dermal Tissue Penetration of In-Plane Silicon Microneedles Evaluated in Skin-Simulating Hydrogel Rat Skin and Porcine Skin. Biomater. Adv. 2023, 155, 213659 10.1016/j.bioadv.2023.213659. 37939443

[ref58] Mutlu M. E.; Ulag S.; Sengor M.; Daglılar S.; Narayan R.; Gunduz O. Electrosprayed Collagen/Gentamicin Nanoparticles Coated Microneedle Patches for Skin Treatment. Mater. Lett. 2021, 305, 130844 10.1016/j.matlet.2021.130844.

[ref59] Halder J.; Rath G.; Rai V. K. Cyclosporine Coated Microneedle for Transcutaneous Delivery: Characterization, *in Vitro* Evaluation, and *in Vivo* Anti-Psoriatic Efficacy against IMQ-Induced Psoriasis. Journal of Drug Delivery Science and Technology 2022, 73, 103450 10.1016/j.jddst.2022.103450.

[ref60] Zhang D.; Das D. B.; Rielly C. D. Microneedle Assisted Micro-Particle Delivery from Gene Guns: Experiments Using Skin-Mimicking Agarose Gel. J. Pharm. Sci. 2014, 103 (2), 613–627. 10.1002/jps.23835. 24399616

[ref61] Al-Qallaf B.; Das D. B. Optimization of Square Microneedle Arrays for Increasing Drug Permeability in Skin. Chem. Eng. Sci. 2008, 63 (9), 2523–2535. 10.1016/j.ces.2008.02.007.

[ref62] Yadav P. R.; Han T.; Olatunji O.; Pattanayek S. K.; Das D. B. Mathematical Modelling, Simulation and Optimisation of Microneedles for Transdermal Drug Delivery: Trends and Progress. Pharmaceutics 2020, 12 (8), 693 10.3390/pharmaceutics12080693. 32707878 PMC7464833

[ref63] Baek S.-H.; Shin J.-H.; Kim Y.-C. Drug-Coated Microneedles for Rapid and Painless Local Anesthesia. Biomed Microdevices 2017, 19 (1), 2 10.1007/s10544-016-0144-1. 28070698

[ref64] Rehman N. U.; Song C.; Kim J.; Noh I.; Rhee Y.-S.; Chung H. J. Pharmacokinetic Evaluation of a Novel Donepezil-Loaded Dissolving Microneedle Patch in Rats. Pharmaceutics 2022, 14 (1), 5 10.3390/pharmaceutics14010005. PMC8778454 35056902

[ref65] Matadh A. V.; Jakka D.; Pragathi S. G.; Rangappa S.; Shivakumar H. N.; Maibach H.; Reena N. M.; Murthy S. N. Polymer-Coated Polymeric (PCP) Microneedles for Controlled Dermal Delivery of 5-Fluorouracil. AAPS PharmSciTech 2023, 24 (1), 9 10.1208/s12249-022-02471-x. 36450897

[ref66] Kaushik V.; Keck C. M. Influence of Mechanical Skin Treatment (Massage, Ultrasound, Microdermabrasion, Tape Stripping and Microneedling) on Dermal Penetration Efficacy of Chemical Compounds. Eur. J. Pharm. Biopharm. 2021, 169, 29–36. 10.1016/j.ejpb.2021.09.003. 34508806

[ref67] Nagelreiter C.; Mahrhauser D.; Wiatschka K.; Skipiol S.; Valenta C. Importance of a Suitable Working Protocol for Tape Stripping Experiments on Porcine Ear Skin: Influence of Lipophilic Formulations and Strip Adhesion Impairment. Int. J. Pharm. 2015, 491 (1), 162–169. 10.1016/j.ijpharm.2015.06.031. 26117191

[ref68] Zhao X.; Schaffzin J. K.; Carson J.; Ankrum A.; Dobrzykowski E.; Haslam D. B.; Dandoy C. E.; Setchell K. D. R. Analysis of Chlorhexidine Gluconate in Skin Using Tape Stripping and Ultrahigh-Performance Liquid Chromatography-Tandem Mass Spectrometry. J. Pharm. Biomed. Anal. 2020, 183, 113111 10.1016/j.jpba.2020.113111. 32062012

[ref69] Alvarez-Román R.; Naik A.; Kalia Y. N.; Fessi H.; Guy R. H. Visualization of Skin Penetration Using Confocal Laser Scanning Microscopy. Eur. J. Pharm. Biopharm. 2004, 58 (2), 301–316. 10.1016/j.ejpb.2004.03.027. 15296957

[ref70] Mansoor I.; Lai J.; Ranamukhaarachchi S.; Schmitt V.; Lambert D.; Dutz J.; Häfeli U. O.; Stoeber B. A Microneedle-Based Method for the Characterization of Diffusion in Skin Tissue Using Doxorubicin as a Model Drug. Biomed Microdevices 2015, 17 (3), 61 10.1007/s10544-015-9967-4. 26009275

[ref71] Yeung C.; Chen S.; King B.; Lin H.; King K.; Akhtar F.; Diaz G.; Wang B.; Zhu J.; Sun W.; Khademhosseini A.; Emaminejad S. A 3D-Printed Microfluidic-Enabled Hollow Microneedle Architecture for Transdermal Drug Delivery. Biomicrofluidics 2019, 13 (6), 064125 10.1063/1.5127778. 31832123 PMC6906119

[ref72] Yu Y.-Q.; Yang X.; Wu X.-F.; Fan Y.-B. Enhancing Permeation of Drug Molecules Across the Skin via Delivery in Nanocarriers: Novel Strategies for Effective Transdermal Applications. Front. Bioeng. Biotechnol. 2021, 9, 646554 10.3389/fbioe.2021.646554. 33855015 PMC8039394

[ref73] Hoang M. T.; Ita K. B.; Bair D. A. Solid Microneedles for Transdermal Delivery of Amantadine Hydrochloride and Pramipexole Dihydrochloride. Pharmaceutics 2015, 7 (4), 379–396. 10.3390/pharmaceutics7040379. 26426039 PMC4695825

[ref74] Khan S.; Minhas M. U.; Tekko I. A.; Donnelly R. F.; Thakur R. R. S. Evaluation of Microneedles-Assisted in Situ Depot Forming Poloxamer Gels for Sustained Transdermal Drug Delivery. Drug Delivery and Transl. Res. 2019, 9 (4), 764–782. 10.1007/s13346-019-00617-2. PMC6606675 30675693

[ref75] He M.; Jin L.; Wang F.; Wang X.; You Y.; He H. Simple, Ultrasensitive Detection of Superoxide Anion Radical Mutations in Melanoma Mice with SERS Microneedles. Spectrochimica Acta Part A: Molecular and Biomolecular Spectroscopy 2024, 316, 124292 10.1016/j.saa.2024.124292. 38669980

[ref76] Malatesta D.; Defourny S. V. P.; Di Teodoro G.; Seca F.; Guardiani P.; Martino M.; D’Alterio N.; Petrini A. Morphological and Immunohistochemical Characterization of an Oral Metastatic Carcinosarcoma in a Cat. Journal of Comparative Pathology 2022, 199, 17–22. 10.1016/j.jcpa.2022.09.001. 36265216

[ref77] Chen T. A.; Yang F.; Cole G. M.; Chan S. O. Inhibition of Caspase-3-like Activity Reduces Glutamate Induced Cell Death in Adult Rat Retina. Brain Res. 2001, 904 (1), 177–188. 10.1016/S0006-8993(01)02485-4. 11516428

[ref78] Huang C.; Gou K.; Yue X.; Zhao S.; Zeng R.; Qu Y.; Zhang C. A Novel Hyaluronic Acid-Based Dissolving Microneedle Patch Loaded with Ginsenoside Rg3 Liposome for Effectively Alleviate Psoriasis. Materials & Design 2022, 224, 111363 10.1016/j.matdes.2022.111363.

[ref79] Economidou S. N.; Uddin Md. J.; Marques M. J.; Douroumis D.; Sow W. T.; Li H.; Reid A.; Windmill J. F. C.; Podoleanu A. A Novel 3D Printed Hollow Microneedle Microelectromechanical System for Controlled, Personalized Transdermal Drug Delivery. Additive Manufacturing 2021, 38, 101815 10.1016/j.addma.2020.101815.

[ref80] Souto E. B.; Zielinska A.; Souto S. B.; Durazzo A.; Lucarini M.; Santini A.; Silva A. M.; Atanasov A. G.; Marques C.; Andrade L. N.; Severino P. (+)-Limonene 1,2-Epoxide-Loaded SLNs: Evaluation of Drug Release, Antioxidant Activity, and Cytotoxicity in an HaCaT Cell Line. International Journal of Molecular Sciences 2020, 21 (4), 1449 10.3390/ijms21041449. 32093358 PMC7073088

[ref81] Lee K.; Xue Y.; Lee J.; Kim H.-J.; Liu Y.; Tebon P.; Sarikhani E.; Sun W.; Zhang S.; Haghniaz R.; Çelebi-Saltik B.; Zhou X.; Ostrovidov S.; Ahadian S.; Ashammakhi N.; Dokmeci M. R.; Khademhosseini A. A Patch of Detachable Hybrid Microneedle Depot for Localized Delivery of Mesenchymal Stem Cells in Regeneration Therapy. Adv. Funct. Mater. 2020, 30 (23), 2000086 10.1002/adfm.202000086. 33071712 PMC7567343

